# Sigmar1 ablation leads to lung pathological changes associated with pulmonary fibrosis, inflammation, and altered surfactant proteins levels

**DOI:** 10.3389/fphys.2023.1118770

**Published:** 2023-03-27

**Authors:** Naznin Sultana Remex, Chowdhury S. Abdullah, Richa Aishwarya, Sadia S. Nitu, James Traylor, Brandon Hartman, Judy King, Mohammad Alfrad Nobel Bhuiyan, Christopher G. Kevil, A. Wayne Orr, Md. Shenuarin Bhuiyan

**Affiliations:** ^1^ Department of Molecular and Cellular Physiology, Louisiana State University Health Sciences Center-Shreveport, Shreveport, LA, United States; ^2^ Department of Pathology and Translational Pathobiology, Louisiana State University Health Sciences Center-Shreveport, Shreveport, LA, United States; ^3^ Department of Internal Medicine, Louisiana State University Health Sciences Center-Shreveport, Shreveport, LA, United States

**Keywords:** SIGMAR1, physiological function, lung, fibrosis, inflammation, surfactant protein, biological function

## Abstract

Sigma1 receptor protein (Sigmar1) is a small, multifunctional molecular chaperone protein ubiquitously expressed in almost all body tissues. This protein has previously shown its cardioprotective roles in rodent models of cardiac hypertrophy, heart failure, and ischemia-reperfusion injury. Extensive literature also suggested its protective functions in several central nervous system disorders. Sigmar1’s molecular functions in the pulmonary system remained unknown. Therefore, we aimed to determine the expression of Sigmar1 in the lungs. We also examined whether Sigmar1 ablation results in histological, ultrastructural, and biochemical changes associated with lung pathology over aging in mice. In the current study, we first confirmed the presence of Sigmar1 protein in human and mouse lungs using immunohistochemistry and immunostaining. We used the Sigmar1 global knockout mouse (Sigmar1^−/−^) to determine the pathophysiological role of Sigmar1 in lungs over aging. The histological staining of lung sections showed altered alveolar structures, higher immune cells infiltration, and upregulation of inflammatory markers (such as pNFκB) in Sigmar1^−/−^ mice compared to wildtype (Wt) littermate control mice (Wt). This indicates higher pulmonary inflammation resulting from Sigmar1 deficiency in mice, which was associated with increased pulmonary fibrosis. The protein levels of some fibrotic markers, fibronectin, and pSMAD2 Ser 245/250/255 and Ser 465/467, were also elevated in mice lungs in the absence of Sigmar1 compared to Wt. The ultrastructural analysis of lungs in Wt mice showed numerous multilamellar bodies of different sizes with densely packed lipid lamellae and mitochondria with a dark matrix and dense cristae. In contrast, the Sigmar1^−/−^ mice lung tissues showed altered multilamellar body structures in alveolar epithelial type-II pneumocytes with partial loss of lipid lamellae structures in the lamellar bodies. This was further associated with higher protein levels of all four surfactant proteins, SFTP-A, SFTP-B, SFTP-C, and SFTP-D, in the Sigmar1^−/−^ mice lungs. This is the first study showing Sigmar1’s expression pattern in human and mouse lungs and its association with lung pathophysiology. Our findings suggest that Sigmar1 deficiency leads to increased pulmonary inflammation, higher pulmonary fibrosis, alterations of the multilamellar body stuructures, and elevated levels of lung surfactant proteins.

## 1 Introduction

Sigma 1 receptor (Sigmar1) is a member of the Sigma receptor family and was initially thought of as a subclass of opioid receptor (Martin et al., 1976). Subsequent studies confirmed Sigmar1 as an intracellular chaperone protein encoded by SIGMAR1 gene (Aishwarya et al., 2021), and studies showed Sigmar1 protein had a binding affinity to a diverse class of pharmacological compounds (Su, 1982). Sigmar1 is ubiquitously expressed in different tissue types, including the brain, heart, liver, kidney, various glands, gastrointestinal tracts, skeletal muscles, pancreas, and reproductive organs (Su et al., 1988; Kekuda et al., 1996; Mei and Pasternak, 2001; Pan et al., 2017; Aishwarya et al., 2021). Studies identified several recessive mutations in SIGMAR1, which were associated with several diseases, including amyotrophic lateral sclerosis, distal hereditary motor neuropathy, Silver-like syndrome, and frontotemporal lobar degeneration (Luty et al., 2010; Al-Saif et al., 2011; Prause et al., 2013; Li et al., 2015; Ullah et al., 2015; Gregianin et al., 2016; Horga et al., 2016). Studies also showed the potential roles of Sigmar1 ligands in a variety of neurological diseases, including Parkinson’s disease, Alzheimer’s disease, depression, amnesia, and ischemic brain injury (Mishina et al., 2005; Mishina et al., 2008). In addition, several studies also demonstrated Sigmar1’s cardioprotective roles in animal models of cardiac hypertrophy and heart failure (Bhuiyan and Fukunaga, 2009; Bhuiyan et al., 2010; Bhuiyan and Fukunaga, 2011; Bhuiyan et al., 2013a). In previous studies, we reported that Sigmar1 global knockout mice (Sigmar1^−/−^) developed cardiac contractile dysfunction, cardiac fibrosis, and mitochondrial respiratory defects (Abdullah et al., 2018). It has also been shown that treatment with non-selective Sigmar1 agonist improved cardiac contractile dysfunction and cardiac hypertrophy by activating Akt-eNOS signaling axis in pressure-overload-induced cardiac injury model of rat and mice (Bhuiyan and Fukunaga, 2009; Bhuiyan et al., 2010; Tagashira et al., 2010; Bhuiyan et al., 2011a; Bhuiyan et al., 2011b; Bhuiyan and Fukunaga, 2011; Tagashira et al., 2013). Consistently, this protective effect of Sigmar1 agonist was ablated by co-current treatment with Sigmar1 antagonists (e.g., NE100 and haloperidol) (Bhuiyan and Fukunaga, 2009; Bhuiyan et al., 2010; Bhuiyan et al., 2011a; Bhuiyan et al., 2011b). These studies suggest that Sigmar1 has multiple functional roles in different tissues in preclinical models.

Recently, Sigmar1 protein received considerable attention in coronavirus disease 2019 (COVID-19) research in drug repurposing to treat the severe acute respiratory syndrome coronavirus 2 (SARS-Cov-2). A recent proteomic study showed that SARS-CoV-2’s Nsp6 protein (non-structural protein 6) interacts with the Sigmar1 protein (Hashimoto et al., 2022). Subsequent proteomic/chemoinformatic analysis identified that Sigmar1 ligands might perturb the viral-human interactome (Gordon et al., 2020a). In fact, several Sigmar1 ligands underwent clinical trials as a possible treatment for COVID-19 infection (Hashimoto et al., 2022). However, the molecular and physiological functions of Sigmar1 in the lung remain to be elucidated. Lungs are the primary organs of the respiratory system and are responsible for transferring oxygen from the air to the bloodstream. The presence of Sigmar1 in the lung was first detected in rat (Seth et al., 1997) and later in mouse (Langa et al., 2003) using cloning and transcriptomic analysis. Radiolabeling studies also detected the presence of Sigmar1 in the lung membranes of mice and guinea pig (Maruo et al., 2000). Some other studies also identified the pulmonary presence of Sigmar1 in rodents using radiolabeled ligands *in-vivo* (Collier et al., 2007; Lever et al., 2012; Lever et al., 2014; Lever et al., 2015). Studies suggested Sigmar1 as a potential biomarker for lung cancer diagnosis and therapeutic targets as Sigmar1 levels were elevated in lung cancer in animal models as well as in tumor cell lines (Mach et al., 2013; van Waarde et al., 2015). Sigmar1 ligands showed beneficial effects in ameliorating airway inflammation and improving airway remodeling in an asthmatic mouse model (Jiang et al., 2022). In this study, Sigmar1 level was decreased in OVA-induced asthma model, and activation of Sigmar1 by treatment with agonist (i.e., PRE084) improved asthmatic symptoms and reduced the levels of inflammatory cytokines in the airway (Jiang et al., 2022). All these studies together indicated an important role of Sigmar1 in association with pulmonary pathology and demonstrated the effects of pharmacological ligands of Sigmar1 in lung pathology. However, none of these studies demonstrated Sigmar1’s direct physiological function in pulmonary system.

The purpose of the present study was to determine the expression of Sigmar1 protein in the lungs using immune-histochemical and biochemical experiments. We also aimed to elucidate the pathophysiological function of Sigmar1 in the lung using Sigmar1 null mice. We wanted to determine the histological, ultrastructural, and biochemical changes associated with Sigmar1 ablation in mice over aging.

## 2 Materials and methods

### 2.1 Animals and human lung tissues

The global Sigmar1 knockout (Sigmar1^−/−^) mice were obtained from the Mutant Mouse Resource Regional Center in a mixed background (C57BL6×129S/SvEv) and backcrossed with C57BL6 for 10 generations (Bernard-Marissal et al., 2015; Abdullah et al., 2018). Both male and female mice of 2 years of age were used for all the experiments to understand the pathophysiological roles of Sigmar1 protein in mice lungs. All mice housed in cages were accommodated in a well-controlled environment with a 12-h light-dark cycle and provided with regular chow diet *ad libitum* food and water. All animals were cared for and handled according to the *Guide for the Care and Use of Laboratory Animals* (Council, 2010). The Institutional Animal Care and Use Committee at LSUHS-Shreveport approved all experimental procedures and protocols for using animals. The formalin-fixed human lung tissues were obtained from individuals with no clinical signs of lung diseases during autopsy in collaboration with the Department of Pathology and Translational Pathobiology at LSU Health Sciences Center-Shreveport. Human lung tissues used in this study were deemed non-human research by the local IRB owing to the exclusive use of *postmortem* samples.

### 2.2 Tissue histology

Lung tissues were isolated from deeply anesthetized Sigmar1^−/−^ and Wt mice and immediately fixed in 10% buffered formalin, processed, and embedded in paraffin as described previously. The formalin-fixed human lung tissues were obtained from a collaborator and a co-author of this manuscript (James Traylor), which were collected from individuals with no clinical sign of lung diseases during autopsy. Paraffin-embedded tissue blocks were cut into 5 µm serial sections and used for different types of histological staining. 5 μm thin sections were deparaffinized, rehydrated, and stained for Hematoxylin and Eosin (H&E) using H&E staining kit (H-3502, Vector Laboratories) following the manufacturer’s protocol. Picrosirius red and Masson’s trichome staining were used to assess the deposition of collagen content in the lung tissue and determine the fibrotic area, respectively (Molnar et al., 2017). All stained tissue sections were imaged using an Olympus BX40 brightfield microscope using ×10 objective lens in a blinded investigator manner. The percentage of collagen content was measured from Picrosirius red staining (stained red), and the fibrotic area was measured from the Masson’s trichrome staining (stained blue) using National Institutes of Health (NIH) ImageJ software v1.6.0. (Bethesda, MD) (Bhuiyan et al., 2016; Abdullah et al., 2018). 10–15 microscopic fields of lung tissues were randomly selected for quantifications and at least six mice were used per genotype, including male and female. Images were quantified in an analyst-blinded fashion.

### 2.3 Immunohistochemistry

Paraffin-embedded human and mouse lung tissue sections (5 µm thin) were deparaffinized and rehydrated before staining following the previously described protocol (Abdullah et al., 2020). Briefly, antigen unmasking solution (H-3301, Vector Laboratories, Burlingame, CA) was used to retrieve antigen using a heat-induced manner. Slides were incubated with 0.3% v/v hydrogen peroxide to block endogenous peroxidases, followed by 5% serum (Vector Laboratories, Burlingame, CA) blocking at room temperature. Subsequently, the tissue sections were incubated with primary antibodies overnight at 4°C (Sigmar1 1:100 dilution, 61,994, Cell Signaling Technology, Danvers, MA) and with secondary antibodies at room temperature in a humidified box. The amplified signal was obtained between antigen-antibodies for visualization by using Vectastain Elite Peroxidase Kit (Vector Laboratories, Burlingame, CA) followed by Dako DAB Chromogen system (DAKO, Carpinteria, CA) according to the manufacturer’s protocol. Lung tissue sections were then counterstained with hematoxylin, dehydrated, and mounted using Cytoseal XYL mounting medium (Thermo Fisher Scientific, Waltham, MA). Images were taken using Olympus BX40 brightfield microscope under ×10 objective lens with Olympus cellSens imaging software (Olympus Life Science, Waltham, MA). The DAB-precipitated brown positive areas were considered Sigmar1 positive areas.

### 2.4 Immunofluorescence staining

Paraffin-embedded 5 µm thin tissue sections from human and mouse lung tissues were stained for immunofluorescence microscopy to determine Sigmar1’s presence and expression in lungs, as previously described (Bhuiyan et al., 2013b; Aishwarya et al., 2022). Briefly, the lung sections were deparaffinized, rehydrated, and antigen-unmasked by boiling at 100°C for 30 min with 10 mmol/L sodium citrate buffer (pH 6.0). A signal enhancer was used for better imaging for 30 min on the tissue sections followed by blocking buffer (1% bovine serum albumin, 0.1% fish skin gelatin in cold water, and 1% tween 20 in PBS) incubation for 1 h at room temperature. The blocked lung tissue sections were then incubated with primary antibody overnight at 4°C (Sigmar1 1:100 dilution, 61,994, Cell Signaling Technology, Danvers, MA) in a humid slide stain box. After 16 h, the tissue sections were washed with PBS and incubated with Alexa Flour conjugated secondary antibody (1:100 dilution) for 1 h 30 min at room temperature in the humid chamber. The stained sections were counterstained with DAPI (Invitrogen, Waltham, MA) for 5 min to stain nuclei and mounted using Vectashield Hardest Antifade mounting media for fluorescence staining (H1400; Vector Laboratories, Burlingame, CA). Sections were left in a dark place to dry for at least 4 hours and then imaged using a Nikon A1R high-resolution confocal microscope with Nikon NIS elements software (v.4.13.04) under ×10 objective lens.

### 2.5 Protein extraction and western blot analysis

Total proteins were isolated from the lung tissues of Wt and Sigmar1^−/−^ mice and whole cell tissue lysates were prepared for Western blotting following previously described protocol (Abdullah et al., 2018; Abdullah et al., 2020; Aishwarya et al., 2022). The harvested lung tissues were lysed in an ice-cold lysis buffer called Cell Lytic M (Sigma-Aldrich, St. Louis, MO) supplemented with Complete Protease Inhibitor Cocktail (Roche, Basel, Switzerland) using a bead homogenizer and subsequent sonication of the homogenized material. The lysed cell materials were centrifuged to precipitate insoluble cell debris, and supernatants with soluble proteins were collected. Protein concentrations of lung tissue lysates were measured using the modified Bradford Reagent (Bio-Rad Laboratories, Hercules, CA). An equal amount of proteins were used to prepare samples using 6x Laemmli’s sample buffer for Western blotting. 20–30 µg of proteins were loaded for sodium dodecyl sulfate-polyacrylamide gel electrophoresis (SDS-PAGE) for protein separation according to molecular weights and transferred to polyvinylidene difluoride (PVDF) membrane (Bio-Rad Laboratories, Hercules, CA). The PVDF membranes containing proteins were then blocked using 5% non-fat skim milk and incubated with primary antibodies overnight at 4°C on a rocker at a slow speed. The membranes were then washed with 1x TBS-T buffer and incubated with alkaline-phosphatase conjugated secondary antibodies (Jackson ImmunoResearch Laboratories, Inc., West Grove, PA) for 1 h 30 min at room temperature on the slow rocker. Finally, the antibodies’ probed membranes were developed using ECF substrate (Amersham, Little Chalfont, UK) and imaged on a ChemiDoc™ Touch Imaging System (Bio-Rad Laboratories, Hercules, CA). The scanned images were viewed in Bio-Rad Image Lab software v.6.0.1 and densitometric analysis was performed in NIH ImageJ software v1.6.0 (Bethesda, MD). Ponceau S protein stain dye (Acros Organics, Geel, Belgium) was used on the transferred PVDF membrane to see the transfer efficiency and could be used as a loading control. The primary antibodies used for immunoblotting in this study include-anti-Sigmar1 (1:1000; 61,994, Cell Signaling Technology, Danvers, MA), anti-pNFkB (1:1000; 3033S, Cell Signaling Technology), NFkB (1:1000; 4764S, Cell Signaling Technology), anti-Fibronectin (1:1000; ab2413, Abcam, Cambridge, UK), anti-pSMAD2 ser245/250/255 (1:1000; 3104S, Cell Signaling Technology), anti-pSMAD2 ser465/467 (1:1000; 3101S, Cell Signaling Technology), anti-SFTP-A (1:1000; PA5-79987, Invitrogen, Waltham, MA), anti-SFTP-B (1:1000; PA5-42000, Invitrogen), anti-SFTP-C (PA5-102493, Invitrogen), anti-SFTP-D (1:1000; BS-1583R, Bioss Antibodies, Woburn, MA), and anti-β-Actin (1:1000; sc-47778, Santa Cruz Biotechnology, Santa Cruz, CA).

### 2.6 Transmission electron microscopy

Lung tissues isolated from the Wt and Sigmar1^−/−^ mice were immediately cut into small pieces (1 mm (Su, 1982)), overnight fixed with 3% glutaraldehyde in 0.1 mol/L sodium cacodylate buffer and underwent a post-fixation step using 1% osmium tetroxide (OsO_4_). Subsequently, the tissue pieces were counterstained with uranyl acetate and lead salts and embedded in low-viscosity epoxy resins. The resin-embedded tissue blocks were then microscopically cut into thin sections, stained with toluidine blue staining to visualize the ultrastructural organizations of the sections and confirm the tissue integrity, and finally, imaged under JEOL-JEM-1400 transmission electron microscope (JEOL, Peabody, MA) using an advanced microscopy techniques digital camera (Woburn, MA). Male and female lung tissue sections from both Wt and Sigmar1^−/−^ mice were analyzed in an investigator-blinded manner (N = 1 male and 1 female mice per group).

### 2.7 Statistical analysis and data reproducibility

All *in vivo* experiments were investigator-blinded and animals were randomly selected for each group/genotype. The unbiased data collection was confirmed using a numerical ear tagging system for the animals used in the experiments. All the imaging studies included alphanumerically labeled paraffin blocks, tissue slides, and microscopic images. The individual mouse and image identifier numbers were cross-referenced and matched to proceed with the analysis. All data are presented in box and whiskers plots, where boxes represent inter-quartile ranges, whiskers represent data distribution, and lines represent medians. GraphPad Prism software version 8 (La Jolla, CA) was used for all the data analyses for statistical significance. The data were first tested for normality distribution and the data sets that passed the normality assumption were analyzed using parametric unpaired Student’s t-test for groups of two. However, data sets that failed to pass the normality assumption test, were analyzed using non-parametric Kruskal–Wallis test. A *p*-value of equal or less than 0.05 was considered as statistically significant.

## 3 Results

### 3.1 Expression of Sigmar1 in mouse and human lungs

**FIGURE 1:**
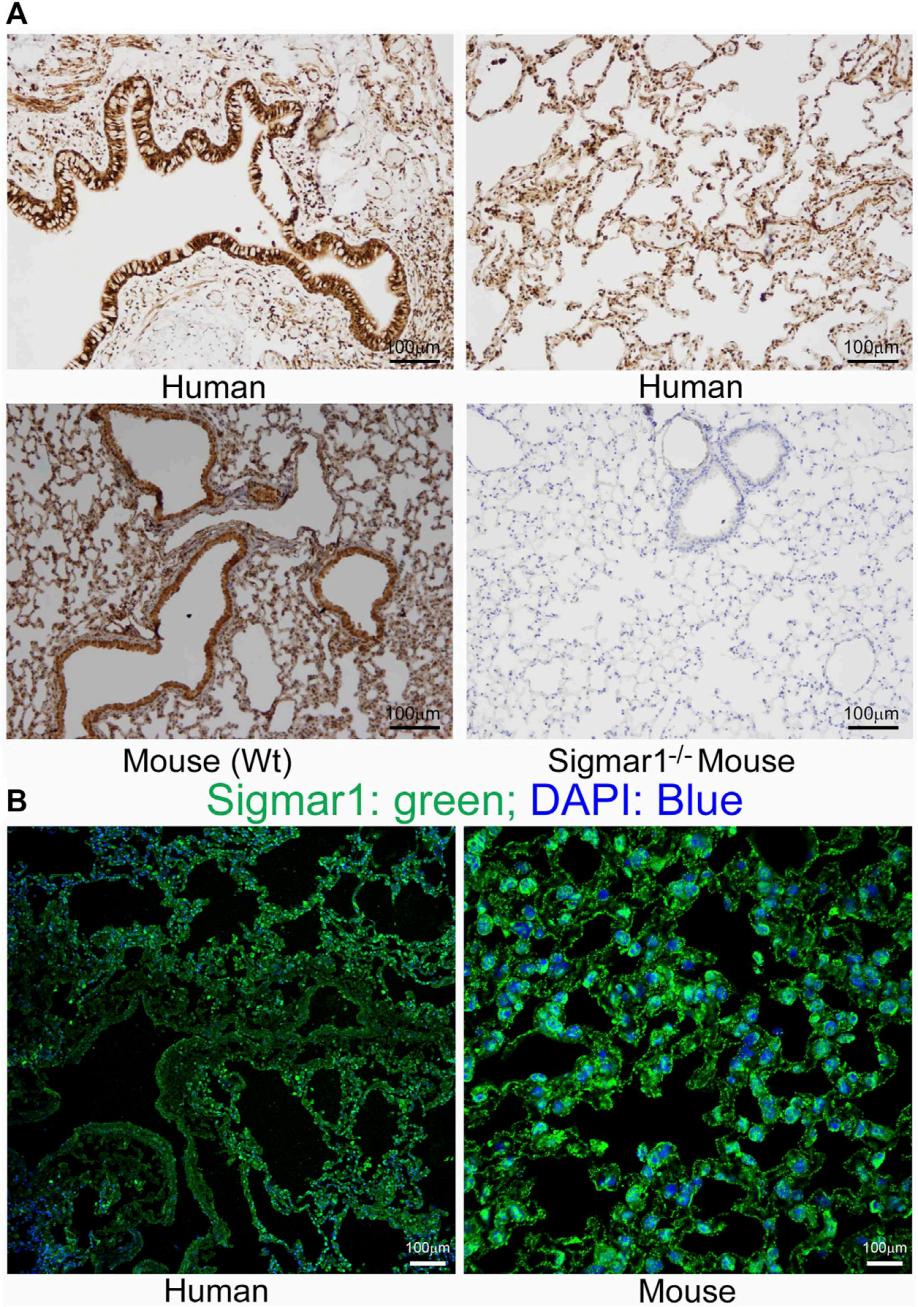
Sigmar1 is substantially expressed in human and mouse lungs. Paraffin-fixed mouse and human lung tissue sections were stained for immunohistochemistry and immunofluorescence. **(A)** Representative images of immunohistochemical staining of human and mouse lung tissue sections with anti-Sigmar1 antibody showed the substantial expression of Sigmar1 in the lung tissues. Immunohistochemical staining of Sigmar1^−/−^ mouse lung was used as a negative control for anti-Sigmar1 antibody and confirmed antibody specificity. **(B)** Immunofluorescence images showed human and mouse lung tissue sections stained for Sigmar1 antibody (green) and counterstained for DAPI as a nuclear stain (blue). Images represent two biological replicates, and 10 to 12 microscopic fields were analyzed. Scale bars: 100 µm.

Sigmar1 is a multifunctional ubiquitously expressed protein that is widely expressed in mammalian cell types (Aishwarya et al., 2021). Several pharmacological studies using ligand binding assays demonstrated the presence of Sigmar1 in the lung (Seth et al., 1997; Maruo et al., 2000; Langa et al., 2003; Collier et al., 2007; Lever et al., 2012; Lever et al., 2014; Lever et al., 2015). However, Sigmar1 expression in the rodent and human pulmonary system has not been well characterized yet. In the present study, we used paraffin-fixed human (with no clinical record of lung disease) and mouse (Wt) lung tissue sections and performed immunohistochemical (IHC) staining using anti-Sigmar1 antibody. The expression of Sigmar1 is determined as brown precipitates, and the sections were counterstained with hematoxylin (blue) for nuclei. The immunohistochemistry staining showed the presence of Sigmar1 in healthy human lung tissue as well as in Wt mouse lung tissues (Figure 1A). IHC staining showed ubiquitous expression of Sigmar1 in all cell types of the pulmonary system and particularly, increased level of Sigmar1 positive staining was observed in the bronchiolar epithelium. A negative control staining of lung tissue from Sigmar1 global knockout mouse (Sigmar1^−/−^) confirmed the specificity of Sigmar1 antibody. Paraffin fixed lung tissue sections were also used for immunofluorescence (IF) staining using anti-Sigmar1 antibody (green), indicating Sigmar1 expression in the human and mouse lung tissues (Figure 1B). Both the IHC and IF staining confirmed the ubiquitous expression of Sigmar1 in the pulmonary systems of human and mouse.

### 3.2 Pulmonary inflammation in aged Sigmar1^−/−^ mice lungs

**FIGURE 2:**
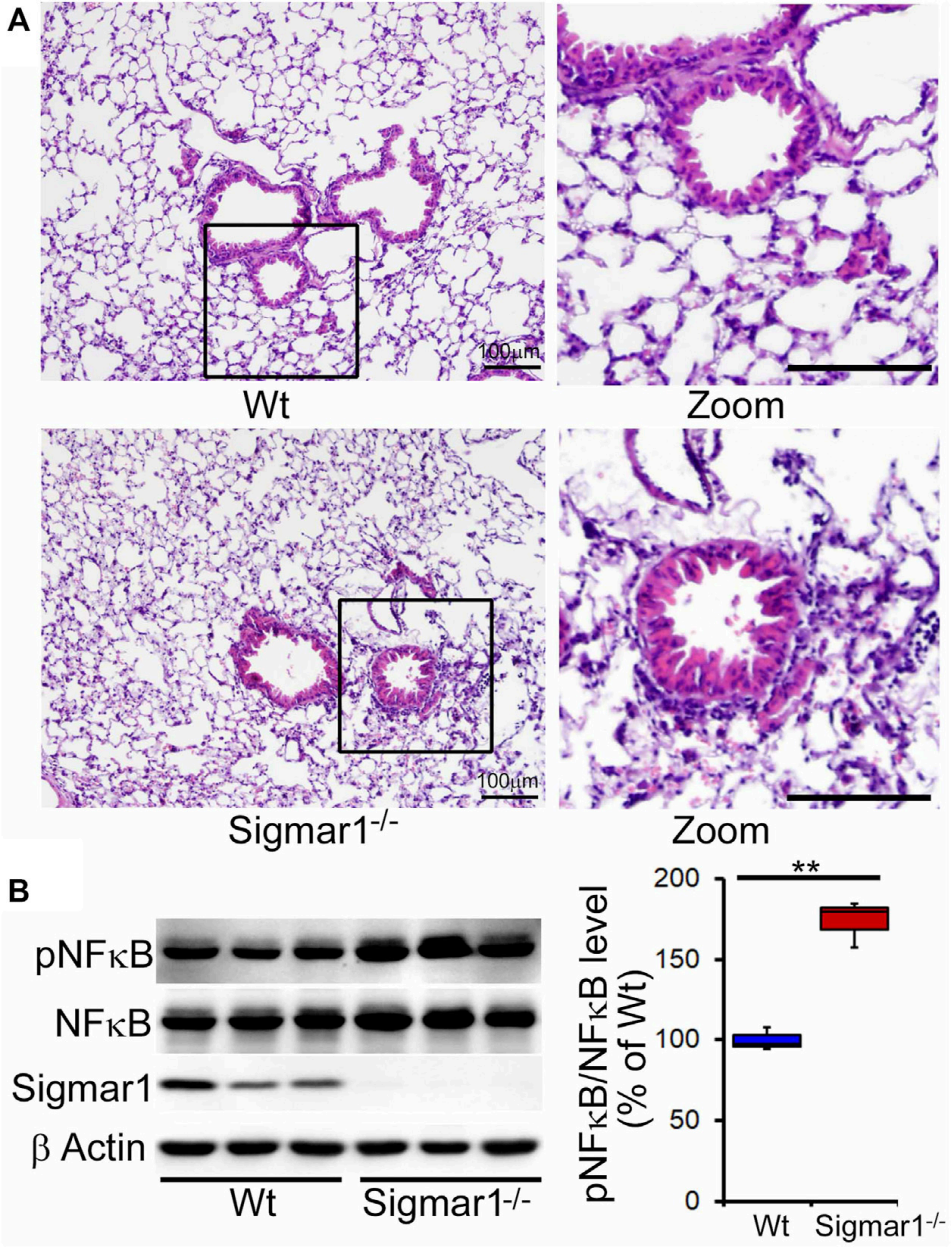
Altered alveolar structures and increased pulmonary inflammation in aged Sigmar1^−/−^ mouse lung. Lungs from aged mice were isolated, and paraffin-fixed tissue sections were stained for hematoxylin and eosin (H&E). **(A)** H&E staining of lung tissue sections showed altered alveolar structures and increased immune cell infiltration in Sigmar1^−/−^ mouse lungs compared to age-match littermate Wt mouse lungs (n = 3 per group). Scale bars: 100 µm; **(B)** Western blot images and quantifications showing increased NFκB phosphorylation in aged Sigmar1^−/−^ mouse lungs compared to age match littermate Wt mouse lungs (n = 3). The Kruskal–Wallis test determined *p* values, and *p < 0.05* was considered statistically significant.

After determining Sigmar1’s expression in the lung tissue, we used Sigmar1 null mouse to determine whether Sigmar1 ablation in mice developed any pathological changes in the lung of the aged mice. Sigmar1^−/−^ mice were reported to have a normal life cycle but were reported to develop several neurological and cardiac pathological changes (Aishwarya et al., 2021). Therefore, we used the aged mice to characterize the lung pathology in the aged mice. Formalin-fixed paraffin tissue sections were used for hematoxylin and eosin (H&E) staining to observe the structural organizations of the lung tissue. H&E staining showed altered alveolar structures, which is the most critical functional unit of lung for exchanging gas, in the absence of Sigmar1 (Figure 2A). Alteration in alveolar structures include not fully open alveoli, pneumocytes disarray, and lots of nucleus infiltration in absence of Sigmar1 in mice. In addition, Sigmar1^−/−^ lungs showed evidence of higher extent of immune cells infiltration indicated by more nuclear staining in purple compared to Wt littermate control (Figure 2A). We have also checked the protein expression level of a very common marker of inflammatory respiratory illness, phosphorylated nuclear factor kappa B (NFkB). The protein expression of phospho-NFkB was significantly higher in Sigmar1 deficient lung lysates (Figure 2B). Lung tissues from three mice per group were used for each experiment. All these data suggested that Sigmar1 null mice develop signs of pulmonary inflammation over aging.

### 3.3 Pulmonary fibrosis in Sigmar1^−/−^ mice lungs

**FIGURE 3:**
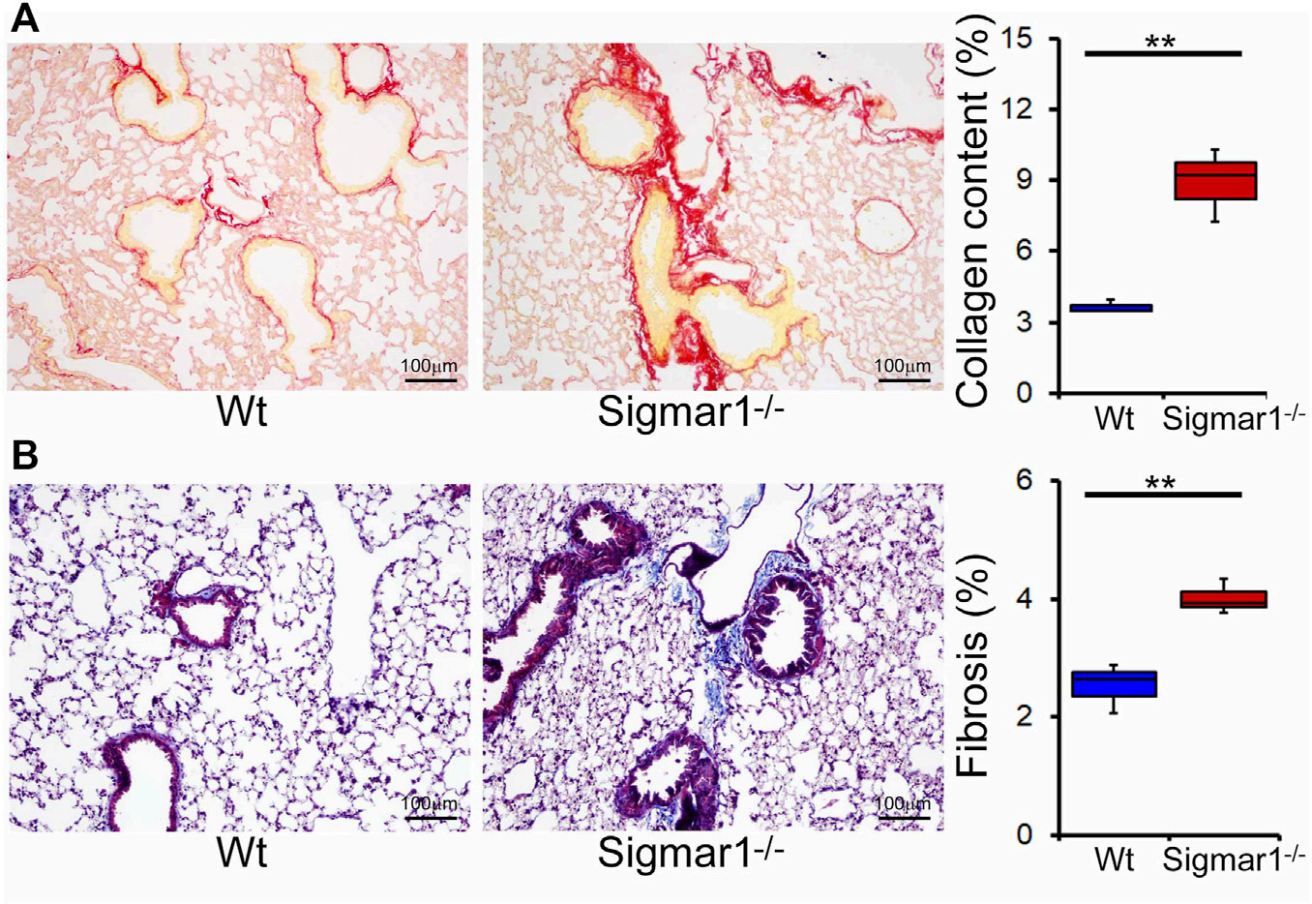
Increased collagen deposition and pulmonary fibrosis in aged Sigmar1^−/−^ mouse lung. **(A)** Picrosirius Red staining of paraffin fixed lung histological tissue sections showed increased collagen deposition (red) in the aged Sigmar1^−/−^ mouse lungs compared to age match littermate Wt mouse lungs (n = 3). **(B)** Masson’s Trichrome staining of paraffin fixed lung histological tissue sections showed increased fibrotic remodeling in Sigmar1^−/−^ mouse lungs compared to age match littermate Wt mouse lungs (n = 3 per group). Scale bars: 100 µm. The Kruskal–Wallis test determined *p* values, and *p < 0.05* was considered statistically significant.

**FIGURE 4:**
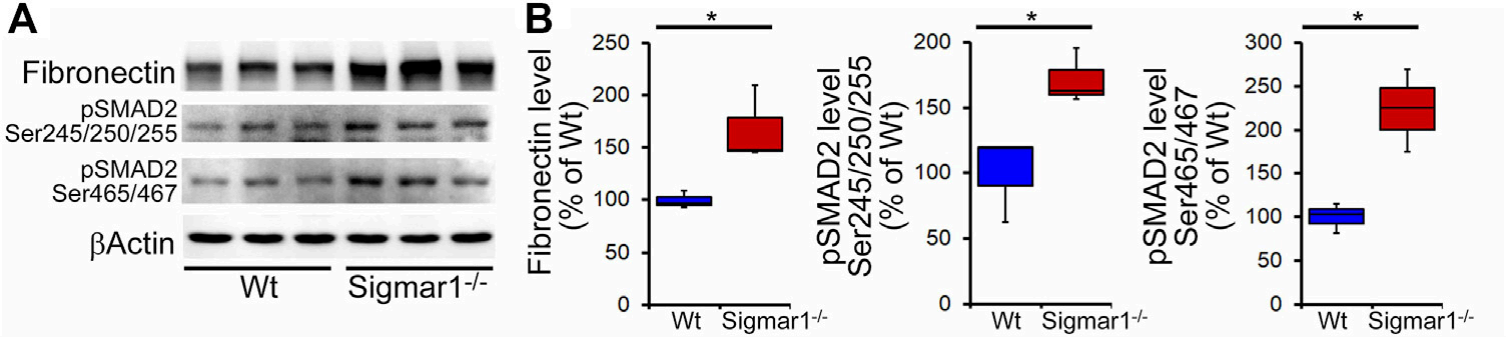
Increased fibrosis regulatory protein level in aged Sigmar1^−/−^ mouse lung. **(A)** Western blot images and **(B)** densitometry analysis showed increased fibronectin, phospho-SMAD2 Ser245/250/255, and phospho-SMAD2 Ser465/467 protein levels in aged Sigmar1^−/−^ mouse lungs compared to age match littermate Wt mouse lungs (n = 3 per group). The Kruskal–Wallis test determined *p* values, and *p < 0.05* was considered statistically significant.

We also determined collagen deposition and fibrotic remodeling of lung tissues in aged Sigmar1^−/−^ and Wt mice using Sirius red and Masson’s trichrome staining of histological lung tissue sections. Sirius red staining showed significantly increased collagen deposition area, and Masson’s trichrome staining showed significantly higher fibrotic area in the Sigmar1^−/−^ lung sections compared to Wt lung sections (Figures 3A, B). Consistent with this, we also observed an increased expression level of extracellular matrix protein called fibronectin, which is a well-known fibrotic marker, in Sigmar1^−/−^ lung lysates compared to Wt littermate control (Figures 4A, B). As sustained phosphorylation of SMAD2 protein is reported in TGF-β-induced pulmonary fibrosis (Ard et al., 2019), we also measured the protein level of phosphorylated SMAD2 in the lung cell lysates. Sigmar1^−/−^ lung lysates showed significantly increased phosphorylated SMAD2 at serine 245/250/255 and serine 465/467 sites compared to Wt lungs (Figures 4A, B) (n = 3 per group for histological staining and Western blot analysis). All these data together suggest that Sigmar1^−/−^ lungs develop pulmonary fibrotic remodeling over aging.

### 3.4 Ultrastructural analysis of lung tissues from Wt and Sigmar1^−/−^ mice

**FIGURE 5:**
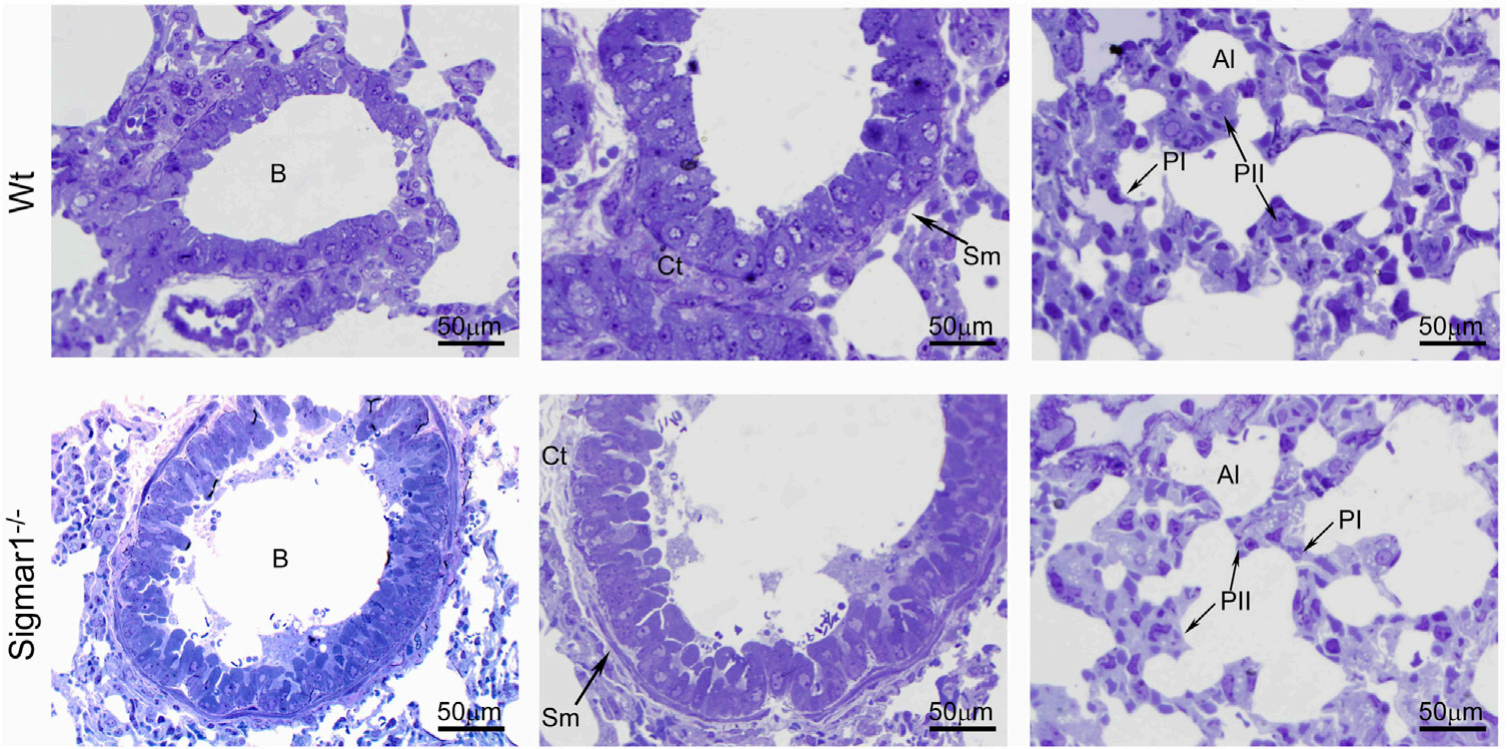
Toluidine blue staining of semithin sections showing the altered alveolar structure in aged Sigmar1^−/−^ mouse lung. Assessment of the Toluidine blue-stained semithin sections showed proper organization of organelles in the aged Sigmar1^−/−^ and Wt mouse lung (n = 3 per group). B = Lumen of bronchioles, Sm = Smooth muscle cells, Ct = Connective tissue, Al=Alveoli, PI = Pneumocyte type I, PII = Pneumocyte type II. Scale bars: 50 µm.

**FIGURE 6:**
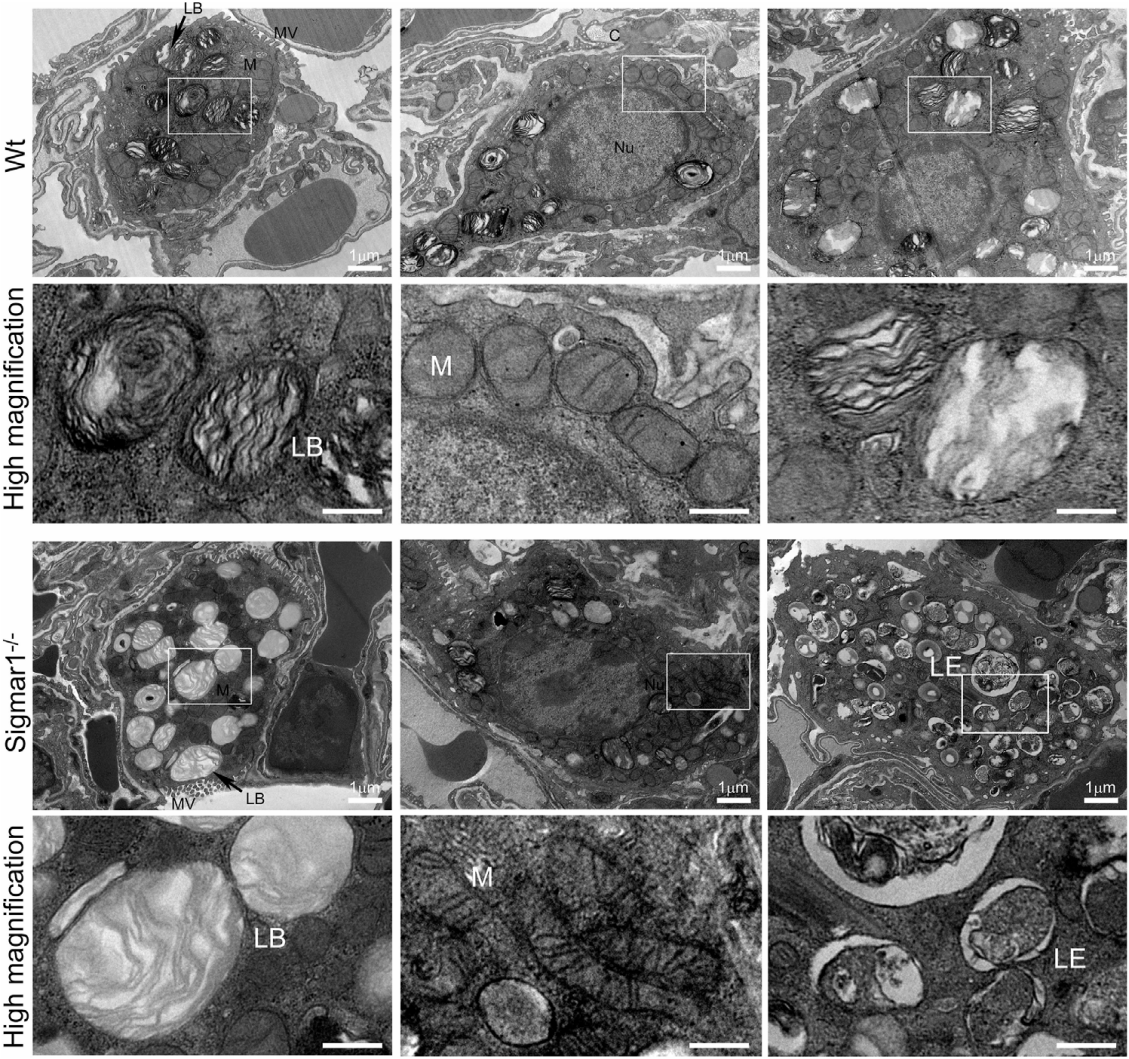
Ultrastructural analysis of type-II alveolar epithelial cell showing altered multilamellar body structure in aged Sigmar1^−/−^ mouse lung. The lung ultrastructure was monitored in the aged Sigmar1^−/−^ and Wt mouse lungs by transmission electron microscopy. Transmission electron micrographs showed the ultrastructural components of Type-II alveolar epithelial cells in Sigmar1^−/−^ and Wt mouse lungs (n = 2 per group). Type-II pneumocytes in Sigmar1^−/−^ mouse lungs showed partial loss of lipid lamellae within the multilamellar bodies. LB = Lamellar bodies, M = Mitochondria, LE = Late endosome, MV = Microvilli, C = Collagen, Nu = Nucleus. Scale bars: 1 µm.

To further understand the direct role of Sigmar1 in respiratory physiology, we have analyzed the ultrastructural components of lung tissue isolated from Sigmar1^−/−^ and Wt littermate controls using transmission electron microscopy (TEM). Toluidine blue staining of semithin sections of the lungs demonstrated the structural organizations of the organelles of lungs including bronchioles (B), alveoli (Al), smooth muscle cells (Sm), connective tissue (Ct), type-I alveolar epithelial cells (PI), and type-II alveolar epithelial cells (PII) (Figure 5). The Sm lining around the B in Sigmar1^−/−^ lungs showed thicker compared to Wt lungs. We performed TEM analysis of these semithin sections for more detailed ultrastructural analysis (Figure 6). The control lungs showed type-II alveolar epithelial cells (PII) with numerous multilamellar bodies (LBs) of different sizes and densely packed lipid lamellae. Interestingly, the transmission electron micrographs of lung tissue sections showed noticeable different structure and pattern of LBs in Sigmar1^−/−^ lungs compared to control Wt lungs. However, PII cells in Sigmar1^−/−^ lungs showed a partial loss in lamellae membranous structures in the lamellar bodies (Figure 6). In addition, Sigmar1^−/−^ lungs also showed accumulation of numerous large endosomes (LE) with scarce amorphous content, elongated mitochondria with comparatively less dense mitochondrial cristae (M), and increased level of collagen (C) deposition (Figure 6).

### 3.5 Altered surfactant protein levels in Sigmar1^−/−^ mice lungs

**FIGURE 7:**
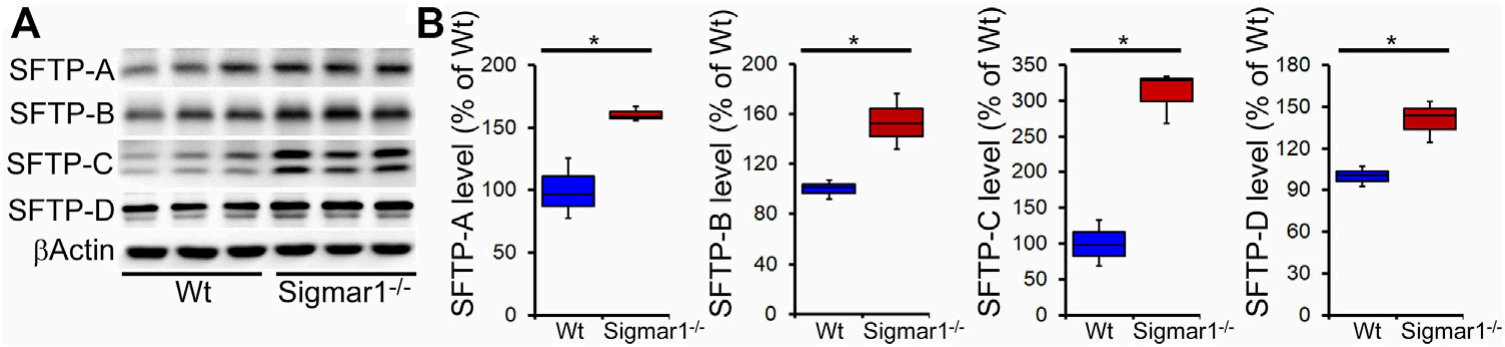
Increased surfactant protein levels in aged Sigmar1^−/−^ mouse lung. **(A)** Western blot images and **(B)** densitometric quantifications showed elevated levels of all four surfactant proteins (SFTP-A, SFTP-B, SFTP-C, SFTP-D) in the aged Sigmar1^−/−^ mouse lungs compared to age match littermate Wt mouse lungs (n = 3 per group). The Kruskal–Wallis test determined *p* values, and *p < 0.05* was considered statistically significant.

Pulmonary surfactant is required to lower the alveolar air-water interface to reduce the surface tension. Surfactant is composed of a unique phospholipid called dipalmitoylphosphatidyl-choline, and four associated proteins, including SFTP-A, SFTP-B, SFTP-C, and SFTP-D (Froh et al., 1990). Most of the surfactant components are stored and released by the multilamellar bodies in the type-II alveolar epithelial cells. Since our data showed an altered structural pattern of lamellar bodies in type-II pneumocytes in Sigmar1^−/−^ mouse lungs, we further investigated if this affected the protein expression levels of the four surfactant proteins. Western blot analysis and densitometric quantification showed the increased protein levels for the SFTP-A, SFTP-B, SFTP-C, and SFTP-D precursor proteins in the aged Sigmar1^−/−^ mouse lungs compared to age match littermate Wt mouse lungs (Figure 7). Lungs tissues were collected from three mice per group for comparing surfactant proteins levels in lungs.

## 4 Discussion

Sigma1 receptor protein expression in rats, mice, and guinea pigs’ lung tissue was confirmed using ligand binding techniques and radiolabeling studies (Seth et al., 1997; Maruo et al., 2000; Langa et al., 2003; Collier et al., 2007; Lever et al., 2012; Lever et al., 2014; Lever et al., 2015). However, these studies could not show Sigmar1’s expression level in these lung tissues. Several ligand dependent studies suggested the potential roles of Sigmar1 ligands in anti-inflammatory, anti-pulmonary remodeling, lung cancer, and alleviating endoplasmic reticulum (ER) stress resulting from viral replication after infection (Mach et al., 2013; van Waarde et al., 2015; Gordon et al., 2020b; Hashimoto et al., 2022; Jiang et al., 2022). To pharmacologically target Sigmar1 in treating pulmonary diseases, it is crucial to understand Sigmar1’s direct physiological functions in the lung. In this present study, we confirmed the expression and localization of Sigmar1 protein in the mouse and human lungs using immunological and biochemical techniques. We found that Sigmar1 deficiency results in pulmonary inflammation, pulmonary fibrotic remodeling, and dysfunction in pulmonary type-II pneumocytes, including altered surfactant protein expression in the lungs of aged mice. This study will form the basis and guide future studies to investigate Sigmar1’s role in pulmonary physiology and pathology.

A previous study showed that Sigmar1’s expression is significantly decreased in the lung tissue of an OVA-induced asthmatic mouse model using immunohistochemical and Western blot analysis (Jiang et al., 2022). They also showed that use of Sigmar1 agonist (PRE084) could restore the SIgmar1 expression while use of an antagonist (BD1047) could inhibit the Sigmar1 expression in OVA-challenged mice (Jiang et al., 2022). In our study, we found the ubiquitous expression of Sigmar1 protein in mice lungs using immunohistochemical staining, immunofluorescence staining, and Western blot analysis. Using the same techniques, we also found Sigmar1 protein expression and localization in healthy human lungs that had no clinical history of lung disease. In this manuscript, for the first time, we determined the expression of Sigmar1 protein in the mouse and human lung tissues. However, any cell-type specific markers have not been used for lung epithelial cells to dissect if there is a different pattern of Sigmar1’s expression depending on lung epithelial cell types.

Ligand-dependent studies showed the anti-inflammatory effects of Sigmar1 activation using its agonist, fluvoxamine, in inflammatory bowel disease (Almasi et al., 2020). Fluvoxamine also showed beneficial anti-inflammatory effects in the preclinical model of septic shock or acute inflammation (Rosen et al., 2019). Activation of Sigmar1 using fluvoxamine lowered the levels of inflammatory cytokines in systemic circulation and improved survival in septic mice (Rosen et al., 2019). In this study, the Sigmar1^−/−^ mouse lungs showed altered alveolar structures and higher immune cell infiltration indicated by more purple nuclei in H&E staining of lung tissue sections compared to Wt mice lungs. This finding was consistent with the significantly elevated protein level of phosphorylated NFkB of p65 subunit in Sigmar1^−/−^ mouse lungs. Transcription factor NFkB is a master-regulator for the inflammatory signals and has critical roles in inflammatory diseases including atherosclerosis, cancer, neurodegeneration, and auto-immunity (Christian et al., 2016). These results suggest the potential role of Sigmar1 in pulmonary inflammation, and it can be an attractive therapeutic target for acute and chronic pulmonary inflammatory diseases. Consistent with our data, a previous study revealed that using Sigmar1 agonists can alleviate pulmonary inflammation in mouse OVA-induced asthma models. Similarly, inhibition of Sigmar1 by its antagonist can induce airway inflammation in the asthmatic model (Jiang et al., 2022). Overall, our findings of altered alveolar structures and increased pulmonary inflammation in the absence of Sigmar1 further added significance to studying this protein in inflammatory diseases in the future.

Pulmonary inflammation is closely associated with pulmonary fibrosis and lung injury as per histological analysis of lung tissue from patients with lung fibrosis (Ward and Hunninghake, 1998). Chronic lung inflammation may lead to an incontinent healing response causing excessive deposition of extracellular matrix substances, such as collagen and fibronectin, between the alveoli in the lung, which results in pulmonary fibrosis. Sigmar1^−/−^ mice lungs showed significantly increased collagen deposition and fibrotic area in peri-bronchiolar and peri-vascular region, confirmed by Sirius red and Masson’s trichrome staining. We also observed upregulation of extracellular matrix protein fibronectin and phosphorylated SMAD2 at serine 245/250/255 and serine 465/467 sites. Literature suggests that both SMAD2 and SMAD3 play a critical role in regulating TGFβ1-induced collagen deposition and fibronectin upregulation in pulmonary epithelial cells during epithelial to mesenchymal transition (Mishra et al., 2008; Kolosova et al., 2011; Liu and Shang, 2020; Xia et al., 2021). Extensive studies indicate that phospho-SMAD2 activation is associated with TGFβ1-induced fibrosis, and its expression is inhibited in TGFβ1-induced 16HBE (lower airway epithelial-like cells) cells upon Sigmar1 overexpression (Jiang et al., 2022). Higher collagen deposition around the type-II pneumocytes is also visible in our transmission electron micrographs in the absence of Sigmar1, indicated by the deposition of dot-like structures. Overall, our data suggest that deletion of Sigmar1 leads to higher pulmonary inflammation and associated pulmonary fibrosis as an adaptive response.

Transmission electron micrographs are great ways to see tissue ultrastructure and reveal organelles’ organization and ultrastructural patterns in a particular tissue. The TEM images showed altered LBs structures with partial loss of lamellae membranous structures in type-II alveolar epithelial cells in the absence of Sigmar1. The lung comprises two types of alveolar epithelial cells: type-I and type-II pneumocytes. Type-I cells are squamous epithelial cells that lack most organelles having multiple cytoplasmic plates and are responsible for gas exchange in the alveoli. Type-II pneumocytes, on the other hand, are cuboidal epithelial cells mainly responsible for the production, packaging, storage, and secretion of surfactant proteins to reduce the surface tension in the alveoli. Our TEM images highlighted the type-II pneumocyte. The most striking finding includes an apparent loss of electron-dense, concentric lamellar structures within the LBs in Sigmar1^−/−^ lungs compared to Wt. These altered LBs structures indicate a disruption in surfactant production, processing, or release, which is evident to be associated with cases of respiratory failure and fatal respiratory diseases in the term infants (Cutz et al., 2000; Tryka et al., 2000). Our TEM images also showed altered elongated mitochondrial shape and structure in Sigmar1^−/−^ mice lungs compared to Wt. This finding is consistent with previous studies identifying Sigmar1’s mitochondrial localization and Sigmar1-mediated regulation of mitochondrial function, dynamics, and respiration (Fukunaga et al., 2015; Abdullah et al., 2018; Abdullah et al., 2022).

The most distinctive feature of type II pneumocytes is the presence of the LBs, a membranous structure periodically laminating in vesicles. Most of the lipid composition in MLBs constitutes a neutral phospholipid named dipalmitoyl phosphatidylcholine (DPPC) which also represents the major active component of surfactant (Schmitz and Muller, 1991). LBs in type-II pneumocytes are primarily responsible for synthesizing and secreting pulmonary surfactant proteins to the extracellular surface of lungs to reduce surface tension (Zaitseva, 1978; Kozlov et al., 1984; Rooney et al., 1994). Surfactant proteins are crucial for normal surfactant functions, and defects to LBs may alter surfactant protein levels. The major composition of the lung surfactant is glycerophospholipid (80%), cholesterol (10%), and protein (10%), which may also vary under pathophysiological conditions (Lajoie et al., 2005). The process of surfactant formation, packaging and storage, and releasing from the pneumocyte LBs in type II cells remains mysterious. However, earlier evidence in the literature suggests that surfactant proteins play important roles in the biogenesis of LBs. To date, four surfactant proteins have been identified and characterized in the pulmonary surfactant, including SFTP-A, SFTP-B, SFTP-C, and SFTP-D (Froh et al., 1990). SFTP-A and SFTP-B are responsible for the phospholipid absorption rate to the surface monolayer of type-II pneumocytes (Dobbs et al., 1987; Notter et al., 1987; Wright et al., 1987; Froh et al., 1990). Additionally, SFTP-A and SFTP-D are predominantly present in the lung and maintain surfactant homeostasis. They perform crucial roles in the host-defense mechanism in the lung, hence, are considered valuable biomarkers for several pulmonary diseases (Kuroki et al., 1988; Kuroki et al., 1998; Mason et al., 1998; Kingma and Whitsett, 2006). The surfactant protein levels in the lung, lavage fluid, and serum may vary depending on disease condition and inflammation. In the present study, we found a significant upregulation of protein expression levels of all four surfactant proteins in the lung tissue from Sigmar1^−/−^ mice compared to Wt. Elevated levels of SFTP-A and SFTP-D were also observed in the bronchoalveolar lavage fluid (BALF) in animal models of acute lung injury by either instillation of endotoxin or over-exposure to oxygen (Nogee et al., 1989; McIntosh et al., 1996). SFTP-A was found to be increased in tracheal aspirates and sputum of lungs in patients with adult respiratory distress syndrome and cardiogenic pulmonary edema (Shimura et al., 1996). One previous study also showed a striking elevation of SFTP-A, SFTP-B, and SFTP-C genes in type-II pneumocytes in the lipopolysaccharide (LPS) mediated lung injury model of rat (Sugahara et al., 1996). The mRNA levels of all four surfactant proteins, especially SFTP-A, were detected to be high in type-II pneumocytes in the case of lung adenocarcinoma (Tsutahara et al., 1993). Increased SFTP-A protein is secreted in lung adenocarcinoma cell lines (Takahashi et al., 1996). This elevation of surfactant protein levels in the pulmonary type-II pneumocytes can be an early response to lung injury. In our model, we observed increased surfactant precursor protein levels in the lung tissue suggesting an adaptive response of the damaged alveoli or altered alveolar cells to towards the repair process in the absence of Sigmar1. However, further experiments are needed to determine the secretion level of surfactant proteins in the BALF as well as in the serum of these mice. Future studies should be directed at understanding the molecular mechanisms and consequences of the altered LBs and surfactant protein processing in the lungs through Sigmar1 under pathophysiological conditions.

## 5 Conclusion

Overall, our findings revealed that Sigmar1 is expressed in the lung tissues of humans and mice. The absence of Sigmar1 can lead to altered alveolar structures, increased immune cell infiltration, and pulmonary inflammation in aged mice. Sigmar1 deficiency can also cause increased pulmonary fibrosis, altered LB structures in type-II pneumocytes, and elevated protein levels of four surfactant precursor proteins, including SFTP-A, SFTP-B, SFTP-C, and SFTP-D.

## Data Availability

The raw data supporting the conclusions of this article will be made available by the authors, without undue reservation.
